# Plasma and Intracellular Concentrations of Doxycycline and Azithromycin in Patients with Severe Scrub Typhus

**DOI:** 10.3390/antibiotics15050450

**Published:** 2026-04-30

**Authors:** Debasree Kundu, Merylin Sebastian, Shadab Ahmad, Sohail Khan, Divya Dayanand, Blessed Winston Aruldhas, Binu Susan Mathew, Karthik Gunasekaran, Nalini Newbigging, Kundavaram P. P. Abhilash, Anand Zachariah, Ramya Iyadurai, Samuel George Hansdak, Sowmya Sathyendra, Thambu David Sudarsanam, Abi Manesh, John Victor Peter, Jeanne Salje, Ooriapadickal C. Abraham, Nicholas P. J. Day, Joel Tarning, George M. Varghese

**Affiliations:** 1 Department of Infectious Diseases, Christian Medical College, Vellore 632004, Tamil Nadu, India; debasreekundu@gmail.com (D.K.);; 2 Center for Cellular and Molecular Platforms, Bengaluru 560065, Karnataka, India; 3 Department of Pharmacology, Christian Medical College, Vellore 632004, Tamil Nadu, India; 4 Department of Medicine, Christian Medical College, Vellore 632004, Tamil Nadu, Indiahansdaksg@cmcvellore.ac.in (S.G.H.);; 5 Department of Emergency Medicine, Christian Medical College, Vellore 632004, Tamil Nadu, India; 6 Department of Critical Care, Christian Medical College, Vellore 632004, Tamil Nadu, India; peterjohnvictor@cmcvellore.ac.in; 7 Mahidol Oxford Tropical Medicine Research Unit, Faculty of Tropical Medicine, Mahidol University, Bangkok 10400, Thailand; 8 Centre for Tropical Medicine and Global Health, Nuffield Department of Medicine, University of Oxford, Oxford OX1 2JD, UK

**Keywords:** doxycycline, azithromycin, scrub typhus, drug distribution, pharmacokinetics, antibiotics

## Abstract

Background/Objectives: Scrub typhus, a life-threatening infection caused by *Orientia tsutsugamushi*, is treated with doxycycline or azithromycin. In severe disease, combination therapy with azithromycin and doxycycline had better clinical outcomes than either drug alone. However, it is not clear what causes the improved efficacy. To understand the same, we examined the plasma concentrations, intracellular concentrations, and efficacy of doxycycline, azithromycin, and both drugs in combination in 51 patients with severe scrub typhus. Methods: A randomly selected subset of adult (>18 years) participants from the INTREST trial (Clinical Trials Registry–India, number CTRI/2018/08/015159), who had been randomized in a 1:1:1 ratio to receive doxycycline, azithromycin, or both drugs, respectively, were included in this study for comparative drug concentration analysis. Blood samples were collected on days 0, 1, 3, and 7 to monitor bacterial load using quantitative polymerase chain reaction (PCR). Five milliliters of sterile blood were collected 3–10 h after the final dose on day 7 for comparative drug concentration measured using high-resolution multiple reaction monitoring. Data were analyzed in GraphPad Prism v.10.0.3. Results: Fifty-one patients (males, 59%; median age, 52 years) were enrolled. Fifteen, seventeen, and nineteen patients received azithromycin, doxycycline, and both, respectively. Doxycycline achieved a median plasma concentration of 1112 (42.51–5697) ng/mL and was undetectable intracellularly. The intracellular concentration of azithromycin (1127 [16.78–19,250] ng/mL) surpassed its plasma concentration (227.1 [48.78–1022] ng/mL). On day 3, PCR negativity rates were 56.24%, 93.3%, and 94.7% in the doxycycline, azithromycin, and combination groups, respectively. Conclusions: The high plasma concentrations of doxycycline and intracellular accumulation of azithromycin may contribute to improved clinical outcomes when used in combination.

## 1. Introduction

Scrub typhus is a life-threatening infection caused by *Orientia tsutsugamushi* and transmitted by larval trombiculid mites. It is a common cause of acute febrile illness in South and Southeast Asia [1]. The primary pathology is characterized by endothelial cell destruction and perivascular leukocyte infiltration induced by *O. tsutsugamushi* that manifests as symptoms ranging from self-limiting febrile illness to severe sepsis syndrome with multisystem organ failure [2].

Doxycycline and azithromycin are effective treatment options for scrub typhus, and early intervention reduces severe complications. A recent randomized clinical trial (the intravenous treatment for scrub typhus—INTREST trial) demonstrated that combination therapy with azithromycin and doxycycline resulted in significantly better clinical outcomes than monotherapy with either agent, without increasing the risk of adverse effects [3]. As compared to doxycycline monotherapy, combination therapy reduced the occurrence of primary outcome events by 13.3 percentage points (47% versus 33%; *p* = 0.002). Likewise, as compared to therapy with azithromycin alone, combination therapy reduced primary outcome events by 14.8 percentage points (48% versus 33%; *p* < 0.001). However, it is not clear what causes the improvement in efficacy.

One cause could be that the two drugs target different sites on the bacterial ribosome. Doxycycline hampers the synthesis of essential bacterial proteins by preventing charged aminoacyl–tRNA from associating with ribosomal A sites, while azithromycin interferes with the 23S rRNA of 50S ribosomal subunits.

Another possibility is that the distribution of the drugs contributes to the synergistic effect. Doxycycline, being a lipophilic drug with a large volume of distribution that binds with plasma albumin (80–90%), has a long terminal elimination half-life of 15–20 h [4]. Azithromycin is also a lipophilic drug and accumulates within white blood cells (WBCs) [5]. Its dibasic structure allows it to be trapped in acidic lysosomes within WBCs [6], resulting in sustained high intracellular drug concentrations long after drug administration [7]. These distributional properties of azithromycin result in widespread tissue distribution and a long terminal elimination half-life of approximately 75 h [8].

This study, which was a subset of the INTREST trial, aimed to measure azithromycin and doxycycline concentrations in plasma and WBCs seven days after initiating intravenous treatment to provide insights into their pharmacokinetic properties and to help generate a deeper understanding of the cause of improved efficacy seen with combination therapy when compared with monotherapy in treating patients with severe scrub typhus.

## 2. Results

Fifty-one patients with a median (range) age of 52 (18–95) years were enrolled (Table 1). Thirty (59%) patients were males. Fifteen, seventeen, and nineteen patients received azithromycin, doxycycline, and both, respectively. The organ systems involved included cardiac (39.2% of patients), respiratory (62.7%), hepatic (47%), renal (35.3%), hematological (13.7%), and central nervous systems (15.7%).

**Figure 1:**
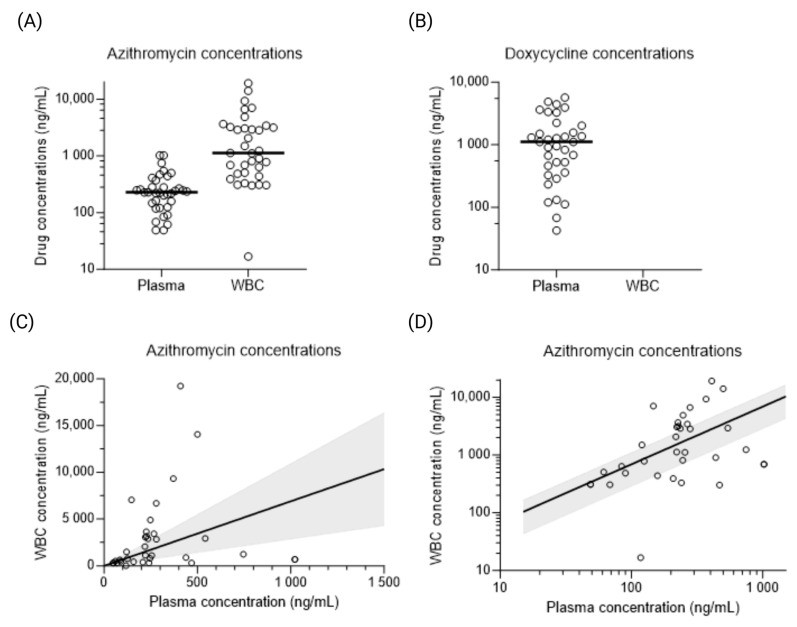
Evaluation of plasma and intracellular concentrations of azithromycin and doxycycline. Group comparison of azithromycin (**A**) and doxycycline (**B**) concentrations in plasma and in white blood cells (WBCs), where horizontal bars represent median drug concentrations. Simple linear regression analysis of azithromycin concentrations in plasma and WBCs, illustrated on a linear scale (**C**) and on a log10-transformed scale (**D**), where the solid line represents the linear regression and the shaded area is the 95% confidence interval of the regression analysis.

The mean duration before fever defervescence was 13 h, 23 h, and 9.7 h in the azithromycin, doxycycline, and combination therapy groups, respectively. The median (range) concentrations of doxycycline were 1112 (42.51–5697) ng/mL in plasma and undetectable intracellularly. The median intracellular WBC concentration of azithromycin (1127 [16.78–19,250] ng/mL) was five-fold higher than its median plasma concentration (227.1 [48.78–1022] ng/mL) (Figure 1A). Plasma and WBC concentrations of azithromycin were significantly correlated (*p* < 0.01), but linear regression analysis showed a large dispersion of data and an estimated slope of 6.89 (95% confidence interval, 2.87–10.9) (Figure 1B).

The baseline PCR results showed that all patients, except one, were positive for *O. tsutsugamushi* (50/51; 98%). Following 3 days of treatment, PCR analysis indicated that 56.3% (9/16), 93.3% (14/15), and 94.7% (18/19) patients tested negative for *O. tsutsugamushi* in the doxycycline, azithromycin, and combination treatment groups, respectively. After 7 days of antibiotics, all treatment groups showed 100% bacterial clearance.

## 3. Discussion

This study confirmed that azithromycin distributes well into WBCs, although its intracellular concentration in our study was lower than previously reported [5]. Contrarily, despite the concentration of doxycycline in plasma being five-fold higher than that of azithromycin, doxycycline was not detected intracellularly, suggesting that doxycycline is either actively removed from cells or not distributed intracellularly at all. The distribution of the drugs could have profound effects on their action and role in bacterial clearance.

The INTREST trial demonstrates the superiority of combination therapy with azithromycin and doxycycline over therapy with either drug alone when treating patients with severe scrub typhus [3]. Another study noted that azithromycin combined with doxycycline was more effective than azithromycin monotherapy for non-gonococcal urethritis [9]. Azithromycin in combination with other antibiotics may also exhibit synergism. In 37 Israeli travelers returning from Nepal with paratyphoid fever, the clinical response to treatment with azithromycin and ceftriaxone was significantly better than that with ceftriaxone alone, with the fever clearance times reduced from six to three days in the combination group [10]. In an RCT of 105 adults with typhoid fever in Nepal, treatment with azithromycin and cefixime for out-patients and azithromycin and ceftriaxone for in-patients had shorter fever clearance times than treatment with azithromycin alone [11].

Our study provides further insights into the effect of combination therapy with azithromycin and doxycycline. The improvement in bacterial clearance with combination therapy as opposed to doxycycline monotherapy and the improved clinical outcomes with combination therapy seen in the INTREST trial may be related to the complementary distribution characteristics of the two drugs. When combined, doxycycline achieves approximately five-fold higher plasma concentrations than azithromycin does, while azithromycin distributes well within WBCs.

*Orientia tsutsugamushi* replicates inside endothelial cells and macrophages and disseminates from infected cells to uninfected cells via an extracellular form that buds off the surface of infected cells [12]. It is possible that doxycycline preferentially targets *O. tsutsugamushi* in the extracellular form while azithromycin targets intracellular organisms. However, this remains to be shown experimentally. Furthermore, the two drugs may target different susceptible strains of *O. tsutsugamushi*. In a previous study, azithromycin was found to be effective against doxycycline-resistant Karp strain rickettsiae and reduced rickettsial growth at 0.0156 μg/mL to levels below that achieved by 0.25 μg/mL of doxycycline [13]. Further studies that investigate the effect of doxycycline and azithromycin on the in vitro susceptibility of *O. tsutsugamushi* are needed.

Three limitations to this study were noted. Firstly, since the intention of the study was to generate a better understanding of what contributes to the efficacy of each drug, the sample size chosen was small. Larger studies are warranted to establish the cause of the improved clinical efficacy of the combination of doxycycline and azithromycin over either drug alone. Secondly, due to logistical and resource constraints, the intracellular concentrations of drugs were estimated only once after the 7-day antibiotic regimen was administered. If WBCs were collected and drug concentrations were measured at multiple points during treatment, it would have provided a more detailed pharmacokinetic profile, particularly for azithromycin. Lastly, the method of estimating the intracellular concentrations can be optimized further in future studies.

In conclusion, based on the results of this sub-study, we hypothesize that when used in combination, the high plasma concentration of doxycycline together with the marked intracellular accumulation of azithromycin may contribute to the improved clinical outcomes reported in the INTREST trial.

## 4. Materials and Methods

This study was a subset of the intravenous treatment for scrub typhus (INTREST) trial, a double-blind, three-arm RCT (Clinical Trials Registry–India, number CTRI/2018/08/015159) that evaluated the efficacy and safety of intravenous doxycycline, azithromycin, or a combination of both drugs in patients with severe scrub typhus [3].

### 4.1. Ethics

Ethical approval was obtained from the institutional review board and ethics committee of Christian Medical College, Vellore (Number: 10493/2017). Participants provided written informed consent. The research was conducted in accordance with the Declaration of Helsinki and national and institutional standards.

### 4.2. Participants

Participants of the INTREST trial underwent block randomization in a 1:1:1 ratio and received doxycycline (200 mg twice daily on day 1 followed by 100 mg twice daily for six days), azithromycin (500 mg twice daily on day 1 followed by 500 mg daily for six days), or both drugs in the mentioned doses, respectively.

A subset of adult patients older than 18 years of age was randomly selected for comparative drug concentration analysis.

### 4.3. Sample Collection

Following the completion of the seven-day intravenous antibiotic regimen, we collected 5 mL of blood in sterile ethylenediaminetetraacetic acid tubes from the participants 3–10 h after they received the last dose of antibiotics. Blood samples were centrifuged to separate plasma from the buffy coat. Plasma samples were stored at −80 °C. Peripheral blood mononuclear cells (PBMCs) were isolated from the buffy coat using Ficoll-Paque density centrifugation as previously described [14]. Cell counts were standardized using a Sysmex CE-2100 hematology analyzer set to 5 × 10^7^ cells/mL. Aliquots of PBMCs were stored at −80 °C.

Blood samples were also collected at baseline, and on days 1, 3, 7, and 14 for quantitative polymerase chain reaction (qPCR) to monitor bacterial load and were stored at −80 °C.

### 4.4. PBMC and Plasma Extraction

PBMCs were washed twice with distilled water and centrifuged at 4000 rpm for 10 min at 4 °C. The pellets were resuspended in a 4:4:2 solution of methanol, acetonitrile, and water, incubated for 30 min at 4 °C, and subjected to nine cycles of sonication for 30 s followed by incubation in ice-water baths. Lysates were further incubated for 30 min at 4 °C and centrifuged at 14,500 rpm for 5 min at 4 °C. Supernatants were stored at −20 °C.

For plasma, 200 µL of the sample was mixed with 800 µL of acetonitrile, incubated at 4 °C for 30 min, and centrifuged at 14,500 rpm for 5 min at 4 °C. Supernatants were stored at −20 °C.

All extracts were dried, reconstituted in 20% acetonitrile with 0.1% formic acid, vortexed, sonicated for 3 min, and centrifuged at 14,800 rpm for 25 min. Samples were diluted in a 1:5 ratio, after which 90 µL of supernatant was spiked with 10 µL roxithromycin (the internal standard). A 10 µL aliquot was injected for LCMS analysis (Figure 1).

### 4.5. Calibration Standards

Stock solutions (5 mg/mL) of doxycycline, azithromycin, and roxithromycin were prepared in water, methanol, and ethanol, respectively. Calibration curves were established for doxycycline (0.01–1600 ng/mL) and azithromycin (0.1–1600 ng/mL), with lower limits of quantification set at 0.01 ng/mL for doxycycline and 0.1 ng/mL for azithromycin. The accuracy ranges were 79–112% and 82–121%, respectively.

### 4.6. Reagents and Instrumentation

Doxycycline hyclate, azithromycin hydrate, roxithromycin, and ammonium acetate were obtained from Sigma-Aldrich, (St. Louis, MO, USA). LCMS–grade acetonitrile and water were purchased from Supelco, (Bellefonte, PA, USA), and Avantor (Radnor, PA, USA), respectively, while formic acid was purchased from Honeywell Fluka (Bengaluru, India).

Chromatographic separation was performed using an LC system (CBM-20A, LC-30AD pumps, CTO-20AC oven, SIL-30AC autosampler) (Shimadzu, Kyoto, Japan) coupled to an Triple-TOF^®^ 5600 mass spectrometer (AB Sciex, Ontario, Canada). Data acquisition and processing utilized Analyst TF 1.6 and MultiQuant 2.1.

### 4.7. Chromatographic Conditions

Analytes were separated on a Luna C18(2) column (150 × 4.6 mm, 5 μm, 100 Å) (Phenomenex, CA, USA). Mobile phase A consisted of 10 mM ammonium acetate with 0.1% formic acid, and mobile phase B consisted of acetonitrile with 0.1% formic acid. A gradient elution was applied (0–2 min: 5% B; 2–7 min: 80% B; 7–7.5 min: 95% B; 7.5–12 min: 95% B; 12–12.1 min: 5% B; 12.1–18 min: 5% B) at 0.6 mL/min.

### 4.8. Mass Spectrometry and Quantification

We optimized the liquid chromatography using tandem mass spectrometry-based high-resolution multiple reaction monitoring (MRM^HR^) quantitation workflow for doxycycline and azithromycin. The collision energy was optimized for selected transitions to achieve optimal signal intensity and selectivity. We employed an MRM^HR^ approach to allow for the simultaneous analysis of drugs and the internal standard while maintaining adequate scan time for reproducible peak integration and quantitation [15]. The total concentration of azithromycin in WBCs was calculated according to Equations (1) and (2) [5].

(1) $$Total\;cell\;volume\;\left(\mu L\right)=Number\;of\;cells\;in\;pellet\times Average\;cell\;volume\left(\mu L\right)$$

(2) $$WBC\;azithromycin\;\left(\mathrm{ng}/\mathrm{mL}\right)=\frac{azithromycin\;in\;lysate\;\left(\mathrm{ng}/\mathrm{mL}\right)\;\times \;lysate\;volume\;\left(\mu L\right)}{Total\;cell\;volume\;\left(\mu L\right)}$$

All available drug concentrations from the different clinical trial arms were pooled to assess the correlation between the plasma and WBC concentrations. Data analysis was performed in GraphPad Prism v.10.0.3.

### 4.9. Quantitative Real-Time PCR

Bacterial load was quantified by real-time qPCR targeting the *O. tsutsugamushi* 47-kDa gene, using an internally controlled TaqMan assay as previously described [14]. DNA was extracted from the buffy coat using the QIAamp DNA Mini Kit (QIAGEN). Standard curves from serial dilutions were included in each run. Results were expressed as genome copies per milliliter of blood. Samples with cycle threshold (Ct) values above 40 were considered negative, while those below 40 were considered positive.

## Figures and Tables

**Table 1:** Patient characteristics of participants.

Patient Characteristics	Total (*n* = 51)	Azithromycin (*n* = 15)	Doxycycline (*n* = 17)	Combination (*n* = 19)
Age, years	52.1 (18–95)	52.3 (19–95)	42.9 (18–70)	60 (29–93)
Male sex (%)	30 (58.8%)	8 (53.3%)	10 (58.8%)	12 (63.2%)
Duration of illness before admission, days	6.3 (2–15)	6.1 (3–10)	6.2 (2–14)	6.6 (2–15)
WBC count, 109/µL	11.4 (4.3–23.6)	11.0 (4.3–23.6)	12.0 (6.3–17.8)	11.3 (5.4–20.4)
Platelet count, 109/µL	87.1 (3.0–330.0)	100.6 (4.0–167.0)	61.2 (3.0–158.0)	96.2 (6.0–330.0)
Total Bilirubin, mg/dL	2.7 (0.2–10.3)	1.8 (0.5–5.5)	3.3 (0.6–7.4)	3 (0.3–10.3)
Aspartate Transferase, IU/L	169.6 (38–1011)	119.5 (46–267)	258.9 (53–1011)	130.4 (38–313)
Alanine Transferase, IU/L	99.2 (27–329)	84.6 (35–164)	129.4 (32–329)	85.7 (27–202)
Serum Creatinine, mg/dL	2 (0.5–6)	1.9 (0.7–6)	2.1 (0.5–5.5)	2 (0.6–5.9)
Fever defervescence duration (hours)	15 (4–132)	13 (4–88)	23 (4–132)	9.7 (4–32)

## Data Availability

Data can be obtained by email request to bmplii@cmcvellore.ac.in.

## Abbreviations

The following abbreviations are used in this manuscript:

INTREST	Intravenous treatment for scrub typhus
RCT	Randomized clinical trial
WBC	White blood cells
qPCR	Quantitative polymerase chain reaction
Ct	Cycle threshold
PBMC	Peripheral blood mononuclear cells
PBS	Phosphate-buffered saline
MRM	Multiple Reaction Monitoring
